# Assessment of the physicochemical characteristics of by-products of cassava processing and their effects on biodiversity

**DOI:** 10.1007/s10661-025-13951-5

**Published:** 2025-04-10

**Authors:** S. A. Olaniyan, J. B. Hussein, M. O. Oke, B. A. Akinwande, T. S. Workneh, M. Ayodele, I. A. Adeyemi

**Affiliations:** 1 Department of Food Science, Faculty of Food and Consumer Sciences, Ladoke Akintola University of Technology, Ogbomoso, P.M.B. 4000 Oyo State Nigeria; 2 Department of Food Science and Technology, Faculty of Agriculture, Modibbo Adama University, Yola, P.M.B. 2076 Adamawa State Nigeria; 3 Department Food Engineering, Faculty of Engineering and Technology, Ladoke Akintola University of Technology, Ogbomoso, P.M.B. 4000 Oyo State Nigeria; 4 Department of Bioresources Engineering, School of Engineering, University of Kwazulu-Natal, Private Bag X01, Pietermaritzburg, 3209 Scottsville South Africa; 5 International Institute of Tropical Agriculture, Ibadan, PMB 5320 Oyo State Nigeria

**Keywords:** Physicochemical properties, Cassava, Wastes, Cyanide, Rural community

## Abstract

Cassava offers by-products of processing such as peels and effluents, which seldom are disposed of without proper treatments. These by-products are rich in organic matter and cyanogenic compounds, which can be potentially hazardous to the environment. For sustainable waste management and ecological balance, a systematic investigation was carried out to analyse the physicochemical properties of cassava peels and effluents and their effects on biodiversity. Standard methods were utilised to analyse these parameters. The results for the effluents ranged from 3.41–3.81 for pH, 2467.10–3630.97 mg/L for biochemical oxygen demand (BOD_5_), 2715.10–3329.90 mg/L for total solids (TS), 1888.20–2235.00 mg/L for total suspended solids (TSS), 869.00–1244.40 mg/L for total dissolved solids (TDS), 3.64–4.85 ppm for cyanide (HCN), and 0.11–0.21 mg/L for total nitrogen (total N). The chemical composition of the cassava peels showed ranges of 85.64–89.81% for dry matter, 12.00–19.50% for crude protein, 3.70–6.00% for crude fat, 2.67–4.59% for ash, 13.00–15.70% for crude fibre, 4.15–7.89% for sugar, 26.36–44.34% for starch, 11.17–12.87% for amylose, and 0.80–14.90 ppm for cyanide content. The analysis revealed that some of the characteristics of the cassava peels and effluents exceeded the standards set by the Federal Environmental Protection Agency of Nigeria (FEPA) and the World Health Organisation (WHO) for drinking water and aquatic life. This study suggests that waste from these processing centres has contributed to environmental pollution in the surrounding communities. Therefore, effective waste management practices are recommended to prevent further environmental degradation.

## Introduction

Cassava (*Manihot esculenta* Crantz) is a vital staple crop in Nigeria, providing a significant carbohydrate source for millions. It is grown on a large scale in tropical and subtropical zones of sub-Saharan Africa, Southeast Asia, and the Pacific Islands for food security and industrial uses such as animal feed production, consumer products, fermentation industries’ raw materials, or starch sources, among many others. While also being an important livelihood activity for smallholder farmers, Nigeria is the leading producer worldwide (Isiwu et al., 2023). On the other hand, cassava processing generates many wastes like peels and effluents, which are not always correctly utilised or discarded with proper care, giving rise to environmental concerns (Adegoke et al., 2020; Mmereki et al., 2024).

**Fig. 1:**
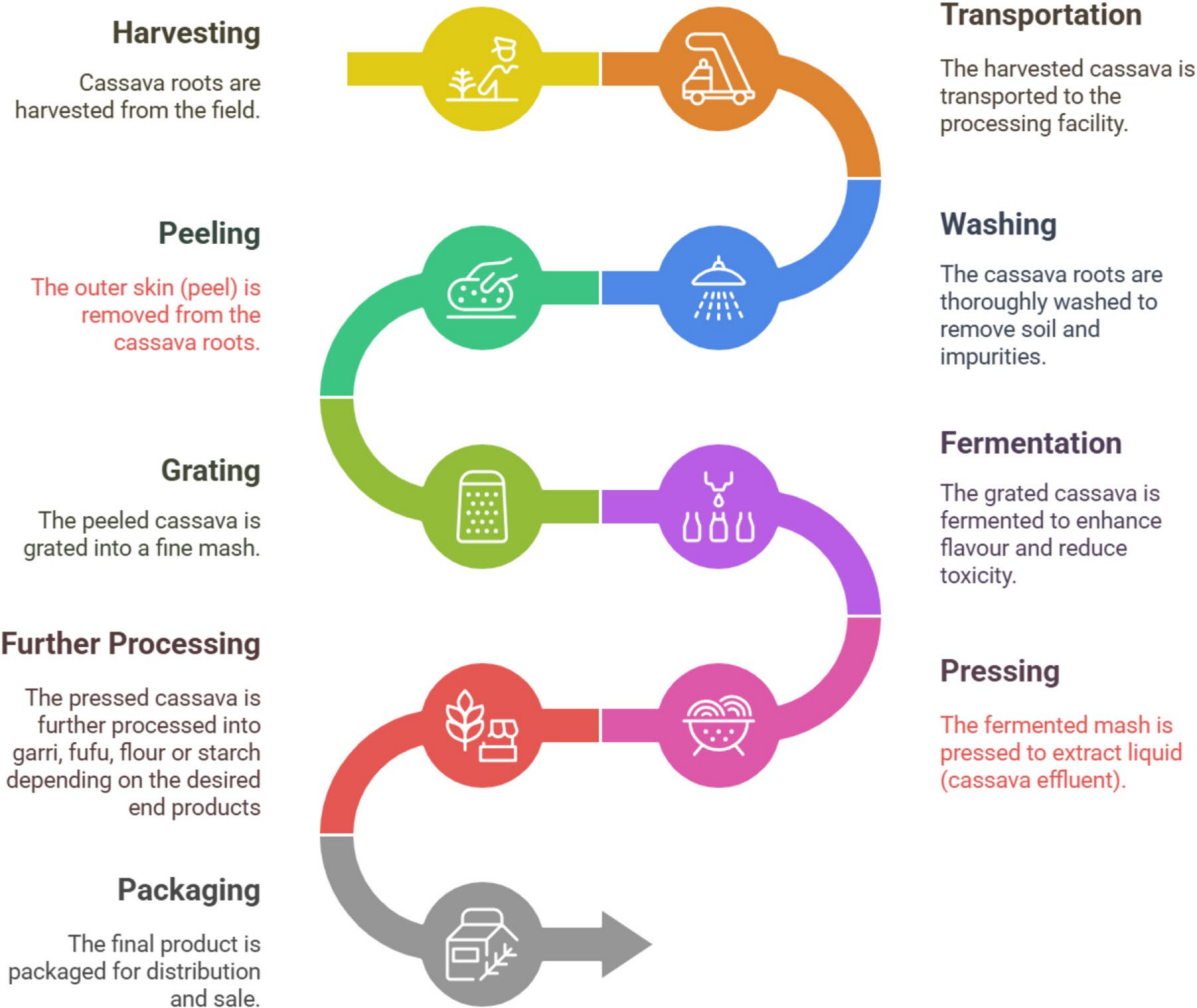
The flow chart for cassava processing that leads to the release of by-products

Figure 1 illustrates the cassava processing that results in the generation of these two primary by-products. The roots are cleaned to eliminate soil and detritus. The external skin of the cassava is excised manually using a knife. The cassava peels are the primary by-products containing cyanogenic chemicals that can be dangerous if not disposed of correctly (Isimah et al., 2023). After washing, the peeled cassava is diced or grated to liberate starch and get it ready for fermentation. Fermenting cassava mash for 1 to 3 days improves texture and decreases cyanide concentration. Once fermentation is complete, the mash is transferred to bags and squeezed dry using a screw press or screw jack. The second by-product is the effluent, which is the wastewater that is produced during the pressing process. Next, the mash is strained to remove excess water. It is then further processed into either *garri*, *fufu*, flour, or starch depending on the target of the processors.

The peel of cassava root contributes about 10–20% to its weight (Adetan et al., 2003). Morgan and Choct (2016) agree that the starchy portion is high in fibre associated with residual starch and can be utilised for animal feed, compost, or other value-added products or byproducts. Rather, the majority of cassava peels are left to decompose, which further increases waste and environmental pollution due to its high-water content that makes it rot fast. These peels, if well used, would reduce the environmental impacts and create new economic opportunities (Oghenejoboh et al., 2021).

Cassava processing generates wastewater containing a variety of contaminants, primarily organic substances and cyanide compounds that are produced most abundantly during washing or fermentation steps. These effluents are a potential health hazard to nearby residents, deteriorate the soil, and pollute available water resources if not treated (Adewumi et al., 2016; Isimah et al., 2023). Cassava effluents are a major environmental problem due to their acidity, high biological oxygen demand (BOD), and total suspended solids (TSS), amongst others, affecting the environment negatively (Oghenejoboh, 2015). Cassava effluents contain elevated levels of organic waste, cyanide, and other contaminants. Upon being released into aquatic environments, they diminish oxygen concentrations, adversely affecting marine organisms (Maciel et al., 2023). Tshala-Katumbay et al. (2016) indicated that exposure to cyanide in cassava effluents may lead to health issues in humans, particularly neurological illnesses. Inadequate disposal of cassava peels results in soil acidification, which adversely impacts plant growth and may endanger both flora and fauna (Amadi et al., 2024; Ben, 2024). Polluted soil and water can diminish crop production and jeopardise food security. Also, effluents contaminating water sources can lead to outbreaks of diarrhoea, cholera, and other waterborne diseases (Otitoju et al., 2024). Kosoe and Ogwu (2024) reported that the decomposition of peels and fermentation of cassava waste release hydrogen cyanide gas, which can cause respiratory problems. Nweke and Onuoha (2024) reported that decomposing cassava peels and effluent release unpleasant odours, contributing to air pollution. The pollution from cassava waste can harm various plant and animal species, leading to a loss of biodiversity (Ujuagu et al., 2025). The emission of pollutants can disturb ecosystem equilibrium, impacting food chains and several ecological processes (Abonyi et al., 2024). Extensive cassava cultivation produces significant quantities of peels, which, if inadequately managed, result in disposal issues and attract pests. This underlies the need for efficient waste management solutions, which should be anchored on a proper knowledge of cassava effluents physicochemical properties.

Cassava peels and effluent may have different physicochemical properties due to the specific conditions as well as processing methods employed in various processing centres. This difference might influence their potential application and environmental impact (Hierro-Iglesias et al., 2024; Idris et al., 2020). Thus, it is very essential to characterise the physicochemical properties of cassava peels and effluents, which are generally discharged by the processing centres into the environment. Their physicochemical properties will ensure to classify them as standardised (safe or unsafe) after applying predefined criteria. In addition, this information is highly useful as organic cultivation systems that have been recently applied to aquaculture (wastewater from cassava processing). The final effluents can as well be used in agriculture lands where non-human food crops are grown. The effluents may be recyclable or could serve as a good soil amendment.

Oyo State in Nigeria ranks among the foremost producers of cassava in the nation. The state’s good soils and favourable climate make it an ideal region for cassava cultivation (Ikuemonisan et al., 2020). Agriculturists in several local government zones engage in both small-scale and large-scale agriculture, guaranteeing a steady supply of this versatile commodity. Ayantoye (2021) indicated that Oyo State has become a dominant force in the cassava value chain, highlighting the state’s abundant agricultural legacy. The cassava processing in Oyo has developed into a dynamic system, considerably contributing to the state’s economy and promoting agricultural sustainability. Therefore, this work investigated the characteristics of cassava peels and effluents from four popular medium-scale processing centres in Oyo State, Nigeria. The centres were situated at Odo-Oba in the ancient town of Ogbomoso; Onipepeye (OIR) located on Old Ife Road, Ibadan; Letmauck Cantonment (LMC), Mokola Barracks in Ibadan; Adekunle Fajuyi Cantonment (AFC) 2 Division Nigerian Army, Odogbo-Ojoo, Ibadan; and Ile Ileri (IIO) in Odo-Oba. These processing centres were selected due to their high production and large processing volume. These results can be used to provide an enhanced understanding of the characteristics and environmental impacts, as well as guide future research on examining waste management and valorisation pathways. The data collected from these processing centres will also help in developing strategies for improving waste management practices and promoting sustainable solutions in the studied area.

## Materials and methods

### Chemicals and sample collections

The chemicals and reagents used were of analytical grade and were acquired from the analytical laboratory at the International Institute of Tropical Agriculture (IITA), Ibadan, Oyo State, Nigeria. The laboratory follows strict quality control measures to ensure accurate and reliable results. All experiments were conducted in accordance with standard operating procedures to maintain consistency and reproducibility. The cassava peels and effluents were collected aseptically at random from the studied processing centres into a sterile plastic container in triplicate. The samples collected were carefully labelled and conveyed to the analytical laboratory of this institute. The analysis was carried out within 24 h after collection in order to avoid pronounced changes in the characteristics of the samples.

### Experimental site

The study was carried out in Oyo State, Nigeria. Oyo State, located in southwestern Nigeria, has a tropical climate marked by pronounced wet and dry seasons (Adeleke, 2019). This climate significantly influences the state’s flora, agriculture, and overall lifestyle. The Wet Season (April to October) is characterised by substantial precipitation, elevated humidity, and verdant flora. During the dry season (November to March), precipitation is scarce, humidity diminishes, and temperatures may increase (Onafeso, 2023). Oyo State endures consistently elevated temperatures year-round, with average daily temperatures fluctuating between 25 °C (77 °F) and 35 °C (95 °F) (Faraday & Oluwabunmi, 2024). The temperature may be marginally elevated in the dry season; however, humidity levels are typically high, particularly in the wet season, resulting in a warm and occasionally oppressive environment. The climate of Oyo State is especially conducive to agriculture. The rainy season supplies sufficient water for crops such as cassava, yam, maize, rice, and vegetables, but the dry season facilitates the production of specific drought-resistant crops such as soybeans, cowpeas, and drought-tolerant maize (IITA, 2024).

**Fig. 2:**
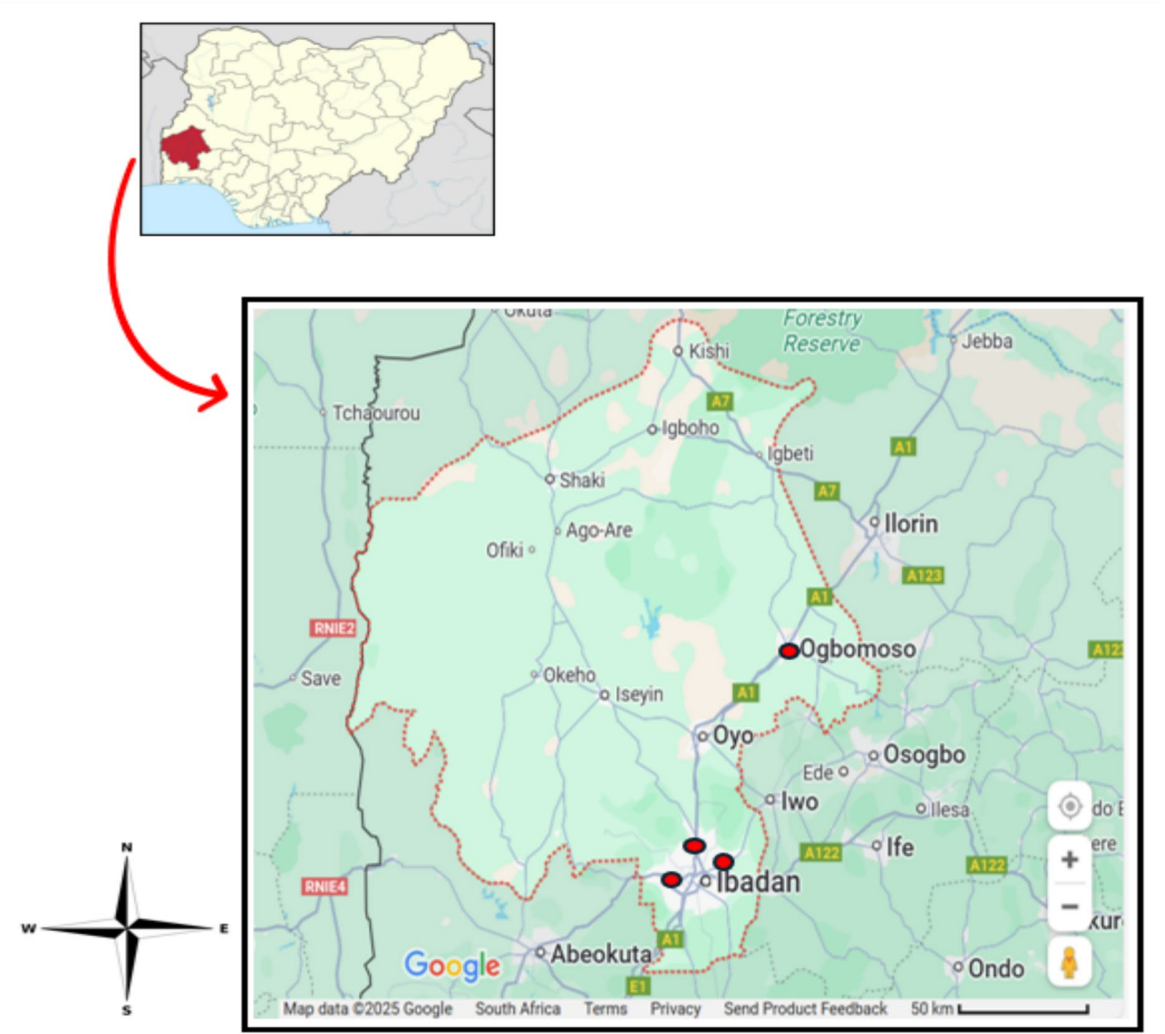
Map showing the study areas in Oyo State, Nigeria. Adapted from Google Map

Figure 2 illustrates the map of the studied areas in Oyo State, Nigeria. Four well-known medium-sized processing hubs in this state were selected for this study. The centres are Adekunle Fajuyi Cantonment (AFC), Odogbo-Ojoo, Ibadan, Letmauck Cantonment, Mokola, Ibadan, Onipepeye, Old Ife Road, Ibadan, and Ile-Ileri in Odo-Oba, Ogbomoso. These processing hubs were decided upon since they processed a very high proportion of the total throughput in the state. These hubs serve as vital points in the agricultural value chain across Oyo State.

### Determination of the physicochemical properties of the cassava processing effluents

The pH, biochemical oxygen demand (BOD_5_), total solids (TS), total suspended solids (TSS), total dissolved solids (TDS), cyanide (HCN), and total nitrogen (total N) of cassava processing effluents were carried out according to the standard methods provided by APHA (2017).

The pH was measured in the effluent sample directly by immersing the electrode and reading it from a calibrated pH meter (Model: EQ- 610 Equip-Tronics). The BOD was determined by incubating a sealed effluent sample with a known amount of dissolved oxygen (DO) at 20 °C for 5 days, after which the decrease in DO was measured to represent the BOD. TS and TSS were determined using the gravimetric method, with results expressed in mg/L. For TS, 50 mL of the effluent was poured into a pre-weighed evaporating dish, evaporated in a water bath, and then dried in an oven at 105 °C until a constant weight was achieved. For TSS, 100 mL of the effluent was filtered through a pre-weighed glass fibre filter, which was then dried at 103–105 °C and weighed to determine the mass increase corresponding to the TSS. TDS were measured by evaporating 10 mL of the filtered effluent sample to dryness and weighing the residue, with results expressed in mg/L. A distillation process and colorimetric analysis were used to measure the cyanide content. The cyanide was turned into hydrogen cyanide (HCN) gas, which was then absorbed in a sodium hydroxide solution and measured using a pyridine-barbituric acid reagent. Total nitrate was measured using a colourimetric method by adding a nitricol tablet to the effluent and monitoring the resulting colour change, which was compared to a standard colour.

### Determination of the chemical composition of the cassava peels

Cassava peels were collected from the cassava processing centres mentioned earlier. The peels from each centre were split into two equal portions. The first portion was fermented for 3 days before being dried in a tunnel dryer at 60 °C, while the second portion was dried at the same temperature in the tunnel dryer without undergoing fermentation. After drying, the peels were milled into flour and the following chemical compositions were analysed using AOAC (2016) standard methods.

The moisture content was measured by drying 2 g of each sample at 105 °C for 3 h using a Fisher Scientific Isotemp Oven (model 655 F, Chicago, USA). Protein content, expressed as % nitrogen × 6.25, was determined using the Kjeldahl method. In this process, 2 g of each sample was digested with concentrated H_2_SO_4_ and a 45% NaOH catalyst. The ammonia released during digestion was distilled into standard boric acid and then titrated with 0.1 M HCl. The total fat content of each sample was determined using the Soxhlet solvent extraction technique. The sample was extracted with ether, and the residue was dried to a constant weight. This weight was used to calculate the percentage of ether extract. The ash content was measured gravimetrically by ashing 2 g of each sample in a clean, pre-weighed crucible. The crucibles were heated in a Fisher Isotemp Muffle Furnace (model 186 A, USA) at 600 °C for 6 h, then cooled to room temperature in a desiccator and weighed. Crude fibre content was determined through an acid-alkaline hydrolysis method. Two (2 g) of the sample were boiled in a beaker with 0.1 M H₂SO₄ and 0.1 M NaOH. The mixture was filtered through a Büchner funnel, and the residue was dried and ashed at 550 °C.

#### Determination of sugar and starch contents

A 0.2 g sample was placed into a centrifuge tube. To this, 1 ml of 100% ethanol, 2 ml of distilled water, and 10 ml of hot ethanol were added. The mixture was vortexed and then centrifuged at 2000 rpm for 10 min. The supernatant, which contains the sugar portion of the sample, was decanted into a separate centrifuge tube. This supernatant was used for sugar determination, while the remaining sediment, which represents the starch portion, was used for starch analysis.

##### Sugar determination

Distilled water (9 ml) was added to the supernatant from the carbohydrate analysis, and the mixture was vortexed. A 0.2 ml aliquot of the vortexed supernatant was pipetted into a test tube. To this, 0.8 ml of distilled water, 0.5 ml of phenol, and 2.5 ml of concentrated H_2_SO_4_ were added and vortexed. The sample was allowed to cool, and its absorbance was measured using a Milton Ray Spectronic 601 Model spectrophotometer, standardised at a wavelength of 490 nm.

1 $$\%\;\mathrm{Sugar}\;\mathrm{content}=\frac{(\mathrm{Abs}\;-\;\mathrm{Intercept})\;\times\;\mathrm{Dilution}\;\mathrm{factor}\;\times\;\mathrm{Volume}}{\mathrm{Weight}\;\mathrm{of}\;\mathrm{sample}\;\times\;\mathrm{Slope}\;\times\;10,000}$$

where Abs = absorbance (abs); dilution factor = 5; volume = 20 ml; slope = 0.0055 and intercept = 0.0044.

##### Starch determination

Approximately 7.5 ml of perchloric acid was added to the starch portion and allowed to stand for 1 h. Then, 17.5 ml of distilled water was added, and the mixture was vortexed. A 0.05 ml aliquot of this solution was pipetted into a test tube. To this, 0.95 ml of distilled water, 0.5 ml of phenol, and 2.5 ml of H_2_SO_4_ were added and vortexed. The sample was allowed to cool, and its absorbance was read using the Milton Ray Spectronic 601 Model spectrophotometer, standardised at a wavelength of 490 nm.

2 $$\%\;\mathrm{Starch}\;\mathrm{content}=\frac{\left(\mathrm{Abs}\;-\;\mathrm{Intercept}\right)\;\times\;\mathrm{Dilution}\;\mathrm{factor}\;\times\;\mathrm{Volume}\;\times\;0.9}{\mathrm{Weight}\;\mathrm{of}\;\mathrm{sample}\;\times\;\mathrm{Slope}\;\times\;10,000}$$

where Abs = absorbance (abs); dilution factor = 20; volume = 25 ml; slope = 0.0055; intercept = 0.0044

#### Determination of amylose and amylopectin contents

A 100 mg sample of cassava peel flour was weighed and placed into a 100-ml volumetric flask. To this, 1 ml of 99.7–100% (v/v) ethanol and 9 ml of 1 N sodium hydroxide (NaOH) were added carefully. The flask was covered with parafilm or foil, and the contents were mixed thoroughly. The sample was then heated in a boiling water bath for 10 min to gelatinize the starch, with the timing starting when the water began to boil. After heating, the sample was allowed to cool completely. This was then made to the reading with distilled water and thoroughly mixed. Then 5 mL of volume of this mixture was taken and transferred to another 100-mL volumetric flask. Then 1 ml of 1 N acetic acid and 2 ml volume of iodine solution were added, followed by topping up to mark with distilled water. At a λ of 620 nm, the absorbance (A) was read with a spectrophotometer.

##### A blank was made for calibration

A total of 1 ml of ethanol and 9 ml of sodium hydroxide were boiled in a flask, which was then filled up to the mark with distilled water. Into a 100-ml volumetric flask, blank prepared in such a way that mix 5 mL of the above solution with 1 l of acetic acid and 2 mL of standard iodine solution; make the volume to mark. This solution was used to normalise at 620 nm using the spectrophotometer.

3 $$\mathrm{Amylose}\;\mathrm{content}\;\left(\%\right)=3.06\times20\times\mathrm A$$

where A = absorbance value

4 $$\mathrm{Amylopectin}\;\mathrm{content}\;\left(\%\right)=100\;-\;\mathrm{Amylose}\;\mathrm{content}$$

### Statistical analyses

All sampling and samples were conducted in triplicate. The collected data were statistically analysed using SPSS software version 22 (Statistical Package for Social Sciences, IBM Corporation, USA). The values were presented as mean ± standard deviation. The means were analysed using the Duncan New Multiple Range Test (DNMRT) at a significance threshold of 5 percent (*p* < 0.05). All graphs were generated utilising Microsoft Excel spreadsheet software, version 2024.

## Results and discussion

### Physico-chemical characteristics of cassava effluents

#### pH level of the cassava effluent

**Fig. 3:**
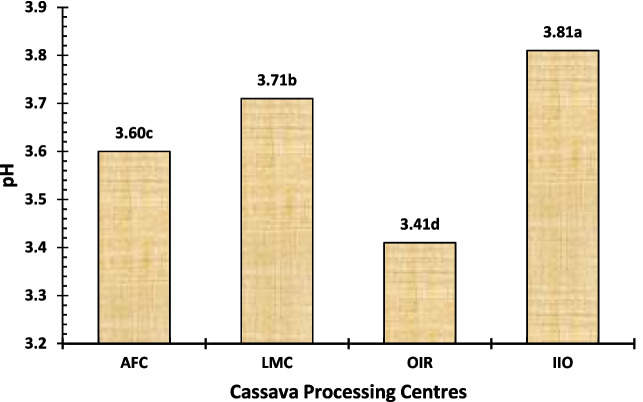
pH level of the cassava effluent

Cassava effluents in this study were acidic, with pH levels ranging from 3.41 to 3.81. The highest pH value was recorded in the IIO processing centre, followed by LMC and AFC, while the lowest were observed with the OIR processing centre, as shown in Fig. 3. These values were below the range of 4.0–4.5, which Okafor (2011) reported for *garri* processing industries at Bida in effluent streams. This disparity in values could be the result of different processing techniques such as cassava varieties, fermentation time, source of water, temperature, environmental conditions, and waste management protocols used by various process centres. Notwithstanding, these pH values are higher than the 6.0 to 9.0 recommended effluent discharge limit stipulated by the environmental regulatory agencies in Nigeria as reported by Bichi (2013) and Kayode et al. (2018). These pH values were also above the range of 6.2–7.3 reported by Koko et al. (2024) for the ground and surface water collected in Oyo state and 6.0–7.0 recommended by WHO (2007) for drinking water. The pH range of 6.5–8.0 was specified by Ayodele and Ajani (1999) for optimum fish production, while Okunle (2004) suggested a higher value between 6.5 and 8.5 as an acceptable level in pond rearing systems habitat water priority constraints. As such, the values obtained in this study area showed that these effluents are acidic as well, which can be due to the presence of hydrogen cyanide and prussic acid in cassava effluent. The findings suggest a threat in terms of effluent that is not at the recommended limits and unsuitable for drinking water consumption or by fish unless properly managed. Moreover, the effluents are acidic and could have consequences for the living conditions of aquatic life and ecosystems in receiving water bodies. Marium et al. (2023) reported that a high pH can cause respiratory distress, ammonia toxicity, and damage to gills, leading to fish kills. Also, a high pH affects fish egg development and hatching success, leading to population decline or loss of biodiversity. Therefore, it is important that the reclamation centre use appropriate processing, such as neutralisation with alkaline or acidic agents and/or biological treatment, to make certain of pH ahead of discharging effluents into the environment. Keeping this clear can help prevent environmental damage, especially for the local wildlife.

#### Biochemical oxygen demand (BOD_5_) level of the cassava effluent

**Fig. 4:**
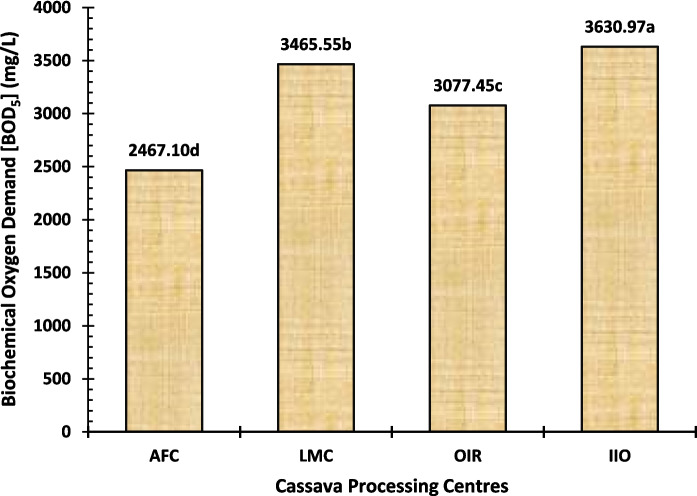
Biochemical oxygen demand (BOD_5_) level of the cassava effluent

BOD_5_ is a parameter that measures the amount of oxygen required by bacteria in order to degrade biodegradable organic material present under aerobic conditions; thus, it represents a key indicator for water quality because it directly reflects all compounds related to the biological load of wastewater (Al-Turki, 2010). The BOD_5_ levels of cassava effluent collected from the processing centres in this study varied between 2467.10 and 3630.97 mg/L; IIO presented higher values compared with LMC and OIR, while AFC was the lowest (Fig. 4). The results showed the IIO processing centre is possibly discharging more organic material into wastewater than other centres. These values are much higher than those reported by Okoye et al. (2023), Okafor (2011), Al-Turki (2010), and Ehiagbonare et al. (2009). They are also above the industrial wastewater FEPA limit of 50 mg/L (Ajibola & Ladipo, 2009). The study of Ehiagbonare et al. (2009) has declared the disposal of cassava effluent in water bodies to be highly hazardous if their BOD_5_ levels are higher, which can have an adverse effect on many aquatic lives, clearly stating that the resulting bastions are not potable. And also, high BOD_5_ is one of the reasons for ammonia and hydrogen sulphide that will be toxic to aquatic ecosystems (Ajibade et al., 2008). High BOD_5_ values indicate a high degree of organic pollution, which poses great risks to flora, fauna, and surfaces as well as groundwater resources (Horsfall et al., 2006; Isabirye et al., 2007). Therefore, it is urgent that these centres make use of efficient BOD_5_ reduction techniques in order to prevent discharging wastewater with high organic loads into aquatic environments. Ignoring this can result in extreme environmental issues, including the destruction of wildlife and likely causing hazards to human life depending on these water sources.

#### Total solids, total dissolved solids, and total suspended solids level of the cassava effluent

**Fig. 5:**
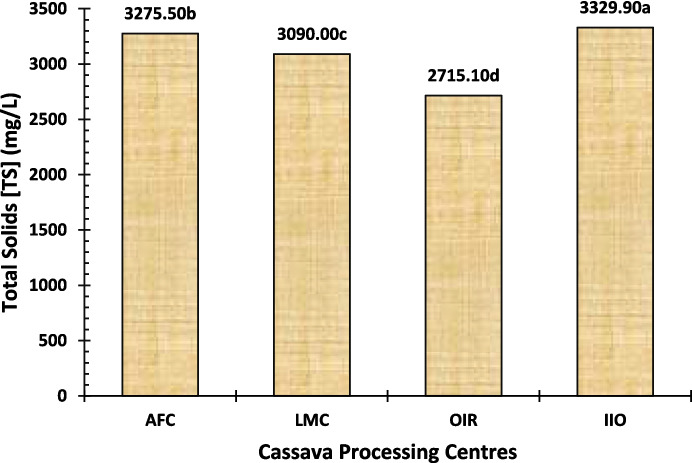
Total solids (TS) level of the cassava effluent

**Fig. 6:**
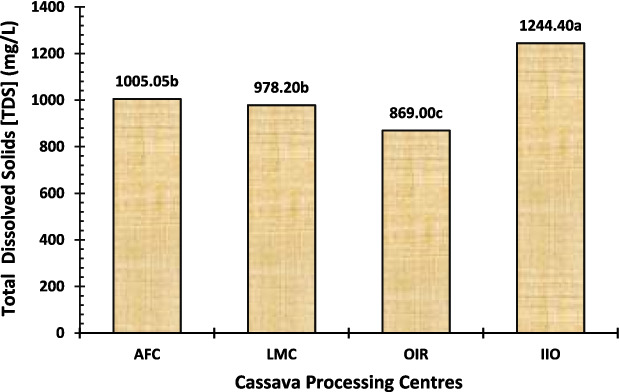
Total dissolved solids (TDS) level of the cassava effluent

**Fig. 7:**
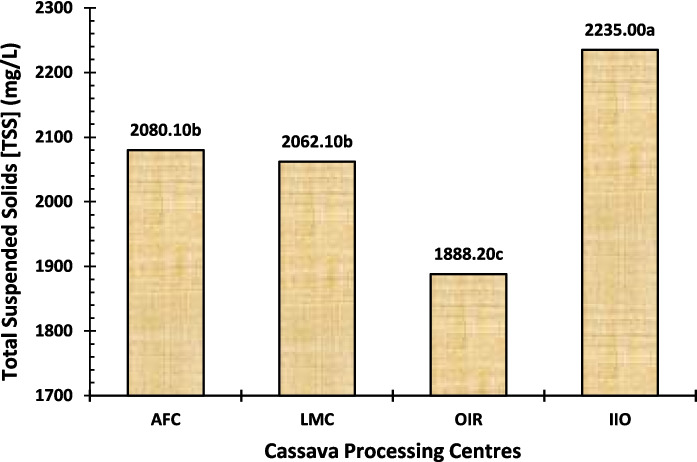
Total suspended solids (TSS) level of the cassava effluent

Total solids (TS) are composed of dissolved solids (TDS) and suspended solids (TSS). The TDS represents the minerals, metals, cations, anions, or salts that are dissolved in water, whereas the TSS is used to quantify how much dry weight of suspended particles (filterable) is present inside the sample. For all the locations evaluated, the TS, TDS, and TSS of waste samples were found to be in the range of 2715.10 − 3329.90 mg/L, 869.00–1244.40 mg/L, and 1888.20–2235.00 mg/L. The TS value was highest at the IIO processing centre, followed by AFC and LMC, with the lowest for the OIR processing centre (Fig. 5). The same trend was observed in the cases of TDS (Fig. 6) and TSS (Fig. 7) among the three study centres. The TSS values were above the WHO and FEPA acceptable limits of 25–30 mg/L, as reported by Sam et al. (2017), Hong et al. (2014), and Okafor (2011). Our findings suggest that effluents from these processing centres may be a source of environmental contamination, as indicated by total suspended solids levels.

In the study by Ehiagbonare et al. (2009), they found that high TSS levels can absorb solar radiation and transfer it to the body, increasing temperature. Among many other effects, this increases water turbidity and decreases light penetration, affecting the local aquatic environment in a detrimental way. The effluents from these centres were usually malodorous, and hence the rank smells are usually perceived with *garri* and *fufu* processing sites as well as environments. When these effluents are discharged without treatment to the environment, it causes severe water pollution. Field observations in the study areas revealed that cassava effluents were generally being discharged directly into rivers and ponds. It can cause water pollution in cracks and damage to aquatic life, as well as a risk to human health if this waste is used for drinking or other purposes. Conclusively, the consequences of cassava processing effluents are environmental and health risks closely linked to waste mismanagement, hence necessitating proper management practices. They should do more to put in place adequate treatment of cassava effluents before discharge, and this includes utilising sedimentation tanks or biological treatment systems. As much as possible, the cassava processing community should be enlightened on the significance of waste management towards promoting sustainable environmental degradation.

#### Cyanide level of the cassava effluent

**Fig. 8:**
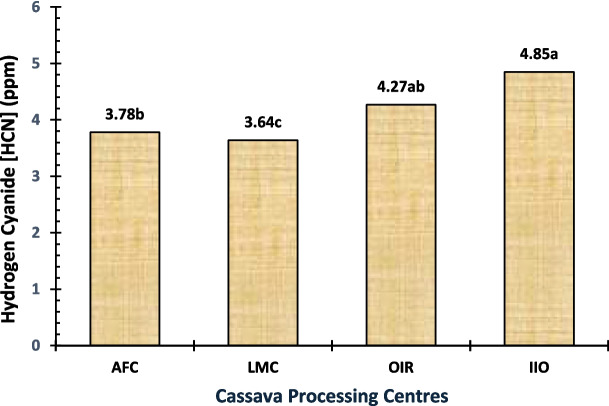
Cyanide level of the cassava effluent

In the cassava effluent, cyanide comes mostly from the degradation of naturally occurring cyanogenic glycosides in cassava. Cyanide can be dangerous to aquatic life and even for people who consume drinking water in which it is present. Cyanide concentrations in cassava effluents depend on the processing techniques and type of cassava used to produce starch. The concentration of cyanide in effluents from processing centres ranged between 3.64 and 4.85 ppm for the IIO that recorded the highest, then OIR and AFC, respectively, but the LMC processing centre had the lowest (Fig. 8). These values are by far greater than the ones reported by Okoye et al. (2023) and Okafor (2011) and also have a concentration greater than 0.07 ppm permitted standard. The considerable cyanide content in the cassava effluents out of processing centres signals an impending environmental and health danger that should be tackled promptly. Efforts should be directed towards reducing cyanide accumulation in cassava processing to help reduce the risks of exposure to high levels of cyanide.

#### The total nitrogen level of the cassava effluent

**Fig. 9:**
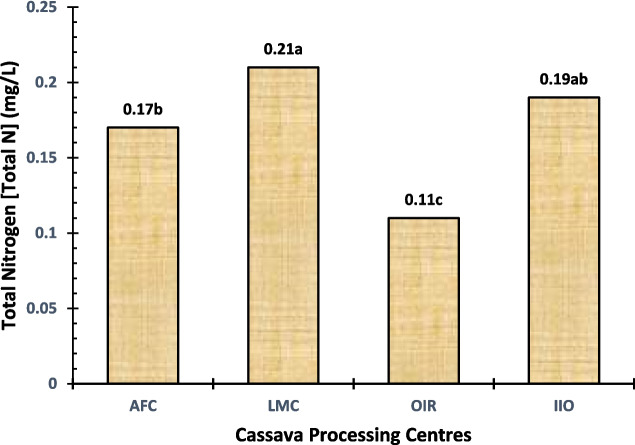
Total nitrogen level of the cassava effluent

Cassava effluent’s total nitrogen is composed of organic and also inorganic fractions such as ammonia and nitrate. Total nitrogen levels in effluents of processing centres ranged from 0.11 to 0.21 mg/L, with the LMC processing centre having the maximum value, while OIR and AFC had almost similar concentrations after that, and IIO was the lowest among all these (Fig. 9). Nutrient pollution occurs when high levels of total nitrogen in effluent reach downstream, such as surface water and subsurface groundwater. This will cause eutrophication as a result of the increased nutrient concentration (Akinnawo, 2023). This process leads to the overgrowth of algae and other aquatic plants that deplete oxygen from water, thus harming aquatic life forms and adversely affecting an ecosystem. Additionally, high nitrate levels in drinking water can lead to potential health consequences such as methemoglobinemia (blue baby syndrome) among infants. Hence, it is crucial to monitor and control the amount of total nitrogen in these factories to avoid potential environmental pollution as well as health hazards. Adoption of efficient treatment processes can improve sustainability and reduce nutrient pollution in water resources, ultimately preserving human health.

#### Comparison of the physicochemical characteristics of the cassava effluents

Table 1 shows the comparison of the physicochemical characteristics of the cassava effluents from the study locations and other processing centres in Nigeria. The results showed variations in all the parameters evaluated among the different cassava processing centres. These differences may be attributed to variations in processing techniques, waste management practices, and environmental factors at each location. Notwithstanding, the values all exceed allowable limits dictated by FEPA/WHO for all categories of industrial effluent to be discharged into the environment. This indicates a potential environmental concern regarding the disposal of cassava effluents from processing centres in Nigeria. Further research and implementation of proper waste management strategies are necessary to mitigate the impact on the environment. Also, there is an urgent need to develop, introduce, and follow up on treatment methods to reduce the potential environmental hazards of this cassava waste before discharge onto agricultural lands.

**Table 1:** Comparison of the physicochemical characteristics of the cassava effluents from the study locations and other processing centres in Nigeria

Study locations	pH	BOD_5_ (mg/L)	TS (mg/L)	TDS (mg/L)	TSS (mg/L)	HCN (ppm)	Total N (mg/L)	References
AFC	3.60	2467.10	3275.50	1005.05	2080.1	3.78	0.17	This study
LMC	3.71	3465.55	3090.00	978.20	2062.1	3.64	0.21	This study
OIR	3.41	3077.45	2715.10	869.00	1888.2	4.27	0.11	This study
IIO	3.81	3630.97	3329.90	1244.40	2235	4.85	0.19	This study
GPI	4.00 − 4.50	511 − 600	420 − 512	237 − 385	183 − 315	0.29 − 0.50	0	Okafor (2011)
CGP	4.10	NA	NA	NA	NA	54.2	NA	Patrick et al. (2011)
SUQ	3.96	NA	5600		NA	685	NA	Olorunfemi and Lolodi (2011)
ECJ	2.50 − 4.20	13 − 73	NA	NA	NA	54.10 − 63.20	NA	Rim-Rukeh (2012)
FAU	5.07	NA	NA	799	789	NA	0.19	Orhue et al. (2014)
LCP	4.20	430.70	NA	975	4030	39	NA	Oghenejoboh (2015)
CAO	6.30	NA	280	NA	NA	1.2	NA	Omomowo et al. (2015)
MFI	3.80	1410	20,619	17,048	3571	0.17	470	Adewumi et al. (2016)
ECA	4.00 − 4.20	1.63 − 2.45	NA	NA	44 − 241	15.12 − 16.86	NA	Sam et al. (2017)
EGA	1.80 − 3.80	58,500 − 76,000	270 − 370	134 − 240	103 − 160	171,110 − 287,040	NA	Okoye et al. (2023)
WHO standard	6.50 − 8.50	10 − 30	2030	2000	25 − 30	0.05 − 0.20	50	WHO (2006)
FEPA standard	6.00 − 9.00	30 − 50	NS	NS	NS	0.1	NS	FEPA (1991)

Where: *AFC* cassava peels from Adekunle Fajuyi Cantonment Processing Centre, Odogbo, Ibadan; *LMC* cassava peels Lekmauck Cantonment Processing Centre, Mokola, Ibadan; *OIR* cassava peels from Old Ife Road Processing Centre, Ibadan; *IIO* cassava peels from Ile Ileri Processing Centre, Odo Oba, Ogbomoso; *GPI* Garri processing industries in Bida, Niger State, Nigeria; *CGP* central Garri processing center at Umuagwo, Imo State, South-eastern Nigeria; *SUQ* small-scale cassava processing mills in Uselu Quarters, Benin City, Edo State, Nigeria; *ECJ* effluent collected from Jesse, a rural community of Delta State, Nigeria; *FAU* Faculty of Agriculture, University of Benin, Benin City, Edo State, Nigeria; *LCP* local cassava processing mill in Oleh, Delta State of Nigeria; *CAO* cassava mills at Arada market located in Ogbomoso, Oyo State, in Southwest Nigeria; *MFI* Matna Foods Industry, Ogbese, Ondo State, Nigeria; *ECA* effluent generated from different hybrid varieties of cassava collected from Akwa Ibom Agricultural Development Program (AKADEP), Uyo, Akwa Ibom State, Nigeria; *EGA* effluent generated form cassava mills in Aba, Abia State, Nigeria; *NA* not analysze; *NS* not specify

### The chemical composition of the cassava peels

Table 2 outlines the chemical characteristics of unfermented and fermented cassava peels. The dry matter fraction of the cassava peels is essentially what remains after all water has been eliminated, and it is an important factor in evaluating their nutrient content, energy value, or any kind of fermentative product that can be generated from this raw material. It is also important for estimating their potential applications, e.g. as animal feed or other wide-ranging biotechnological uses, including agricultural practices. The dry matter content of both the fermented and unfermented samples at all processing centres ranged from 85.64 to 89.81%. These values compare favourably with the 88.58 g/100 g reported by Oladimeji et al. (2022). Fermented cassava peels sourced from the AFC processing centre had a significantly higher value than those processed by LMC, IIO, and OIR centres, which were not different (*p* < 0.05).

**Table 2:** Chemical properties of unfermented and fermented cassava peels collected from the four locations

Sample	Dry matter (%)	Crude protein (%)	Crude fat (%)	Ash (%)	Crude fibre (%)	Sugar (%)	Starch (%)	Amylose (%)	Cyanide (ppm)
UAFC	86.25 ± 0.04^d^	12.40 ± 0.23^ef^	5.70 ± 0.22^a^	9.00 ± 0.31^c^	14.50 ± 0.16^bc^	6.31 ± 0.02^c^	44.34 ± 0.02^a^	11.85 ± 0.02^c^	14.20 ± 0.12^bc^
FAFA	89.81 ± 0.03^a^	12.00 ± 0.06^f^	5.40 ± 0.10^a^	8.90 ± 0.11^c^	14.30 ± 0.12^c^	7.89 ± 0.04^a^	27.36 ± 0.03^c^	11.65 ± 0.02^d^	13.90 ± 0.35^c^
ULMC	85.64 ± 0.02^e^	18.90 ± 0.04^bc^	3.80 ± 0.05^b^	11.40 ± 0.17^ab^	13.30 ± 0.12^d^	4.48 ± 0.03^e^	39.68 ± 0.04^ab^	12.68 ± 0.03^b^	1.00 ± 0.05^de^
FLMC	89.72 ± 0.01^a^	18.50 ± 0.16^c^	3.70 ± 0.08^b^	10.90 ± 0.12^b^	13.00 ± 0.12^d^	6.97 ± 0.04^b^	34.30 ± 0.12^b^	11.17 ± 0.03^g^	0.80 ± 0.01^e^
UOIR	87.62 ± 0.03^bc^	13.20 ± 0.14^d^	6.00 ± 0.39^a^	9.40 ± 0.33^c^	15.70 ± 0.27^a^	4.15 ± 0.02^f^	39.37 ± 5.75^ab^	11.39 ± 0.04^e^	14.90 ± 0.20^a^
FOIR	87.48 ± 0.14^c^	12.60 ± 0.13^e^	5.90 ± 0.29^a^	9.20 ± 0.12^c^	14.90 ± 0.31^b^	4.49 ± 0.03^e^	34.78 ± 0.04^b^	12.87 ± 0.02^a^	14.50 ± 0.14^ab^
UIIO	87.68 ± 0.03^b^	19.5- ± 0.16^a^	4.20 ± 0.12^b^	11.20 ± 0.06^ab^	14.40 ± 0.10^bc^	5.39 ± 0.05^d^	38.49 ± 0.04^ab^	11.91 ± 0.05^c^	1.50 ± 0.09^d^
FIIO	87.66 ± 0.02^b^	19.00 ± 0.17^b^	3.90 ± 0.38^b^	11.80 ± 0.27^a^	13.10 ± 0.06^d^	6.30 ± 0.12c	26.36 ± 0.02^c^	11.28 ± 0.02^f^	1.30 ± 0.03^de^

Mean values with different alphabets in the same column are significantly different at *p* < 0.05

Where: *AFC* cassava peels from Adekunle Fajuyi Cantonment Processing Centre, Odogbo, Ibadan; *LMC* cassava peels Lekmauck Cantonment Processing Centre, Mokola, Ibadan; *OIR* cassava peels from Old Ife Road Processing Centre, Ibadan; *IIO* cassava peels from Ile Ileri Processing Centre, Odo Oba, Ogbomoso; *U* infront of each processing centres stand for unfermented sample, while F stands for fermented sample

On the other hand, the value obtained for the unfermented cassava peels at the IIO processing centre represents the highest, while that of the OIR, LMC, and AFC processing centres values follows, respectively. One of the characteristic changes during cassava peel fermentation is a decrease in dry matter content, with fermented peels generally having slightly higher dry matter contents in comparison to unfermented samples. Dry matter content varies with cassava variety, processing techniques used, and conditions throughout drying. It is important to note that the cassava peel’s ability to serve as raw material in animal feeding or industrial processes greatly depends on its dry matter content. Knowing these differences could help in the optimisation of processing methods, thus enhancing cassava peel utilisation potential across various sectors.

The protein is very important in the diet of an average human for growth and development. The cassava peels ranged from 12.00 to 19.50% in protein content for both unfermented and fermented samples from all locations investigated. Protein content of fermented cassava peels was generally higher than unfermented ones (Table 1). An increase in the crude protein content after fermentation as observed supports work by Oboh (2006), who reported that utilisation of solid media and mixed cultures such as *Saccharomyces cerevisiae* and *Lactobacillus spp*. on cassava peels enhanced the nutrients therein. Morgan and Choct (2016) added that cassava nutritional quality could be increased by gastrointestinal digestibility through supplementally produced protein, or fermenting the interaction between foods containing anti-nutrients as proteins of other ingredients in addition to fermentation may help mitigate effects.

The elevated protein level seen in fermented cassava peels could be due to the extracellular enzymes (proteins such as amylases, linamarase, and cellulase) that are released by microorganisms during fermentation acting on the mashed cassava. The enzymes enhance the proliferation and growth of these organisms; this action leads to the release of more single-cell proteins (Oboh & Akindahunsi, 2003).

The fat content of cassava peels was found to be between 3.70 and 6.00% for the unfermented samples and fermented ones across all locations that were sampled (Table 2). These values are higher than 1.37 g/100 g reported by Oladimeji et al. (2022). The results of this study reveal that there is no significant difference in fat content between fermented and unfermented cassava peels. The fat contents values obtained from all the processing centres were lowered through fermentation; this implies that a slight decrease in fat content could be possible with fermentation treatment but still remain in a low amount. Microbial activity during fermentation is centred on the decomposition of carbohydrates and proteins, as a result in fat content likely changing but not to an appreciable degree. Within the updated fermenting estimates, the fat content in cassava peels remained practically unchanged during fermentation. These results support the possibility of utilising fermented cassava peels as a low-fat alternative in other industrial applications within the food and feed industry.

Ash reflects the inorganic residues left after incineration of organics at 500 to 600 °C. These include traces of minerals such as inorganic soils, salts, silicates, phosphates, and some heavy metals (Enoch et al., 2022; Ibrahim Grema et al., 2023). Ash serves as a surrogate for mineral content, and the ash values in this study were between 2.67 and 4.59%. There was no significant difference (*p* > 0.05) in the ash content of cassava peels when fermented and unfermented. Nonetheless, the ash content of fermented cassava peels was relatively higher than that of unfermented peels (Table 2). This may be a consequence of the conversion/loss of organic matter during fermentation, yielding mineral concentrations. The extent to which this would be necessary would vary depending on the particular fermentation process and conditions.

The value for crude fibre of cassava peels varied between 13.00 and 15.70% in this study from both unfermented and fermented samples, which is evidently higher than 9.40 g/100 g that was reported by Oladimeji et al. (2022). for cassava peel. This high fibre is not much of a surprise because cassava peels are made up of fibrous material that supports the structure of the plant. According to the report of Rogoski et al. (2023) and Zulkifli and Karim (2022), cassava peels have potential as a source of cellulose, hemicelluloses, and lignocellulosic materials suitable for use in various industries, such as animal feeds or separate recovery on biomethane and biogas at landfills. Nevertheless, few authors like Rogoski et al. (2023) and Abotbina et al. (2022), asserted that the fibre amount in cassava peels is lower than other agro-industrial wastes. This high fibre content of cassava peel effluents makes them not safe for human consumption, especially among places where people use cassava as their major food source (Kayiwa et al., 2021). Indeed, peels have a relatively high fibre content, making them difficult to digest (Abotbina et al., 2022). Hence, cassava peels require further uses not intended for human consumption, such as biofuel and compost. In addition, the fabrication of novel technological methods that allow high fibre content in cassava peels to be processed may alleviate environmental challenges linked with the disposal of these wastes.

The sugar level of cassava peels fell within 4.15–7.89% and that for the starch phase diluted from 26.36 to 44.34%. One possible explanation could have been that unfermented cassava peels generally contain low sugar content since simple sugars are not naturally abundant in those types of peel. However, the sugar levels rise after processing due to starch breaking down into simpler sugars enzymatically. The results of the starch content analysis showed that unfermented cassava peels have a higher quantity of starch compared to fermented ones from all processing centres. Cassava is a starchy root crop and, as such, contains starch, which serves as an energy reserve in plants; the absence of enzymatic hydrolysis attributed to fermentation resulted in a higher content of non-reducing sugar (starch) observed in unfermented cassava peels. After they are peeled, the peels still contain residual levels of this starch. This occurs as the microbial enzymes decompose the starch and carbohydrate content of the peel, reducing its total sugar during fermentation due to metabolization by microorganisms. The breakdown of the starches can create many volatile flavour compounds, which are highly important to the eventual taste characteristics of a product once it has been fermented.

The amylose content of cassava peels was between 11.17 and 12.87%. As the natural composition of cassava starch, it is known that amylose generally constitutes around 20–30% in unfermented cassava peels depending on variety and growing conditions (Oladunmoye et al., 2014). During fermentation, starch in cassava peels is acid hydrolysed by endogenous and microbial enzymes that result in reduced amylose content. Amylose, having fewer branches, is more easily hydrolysed in comparison to amylopectin. Amylopectin is the main constituent of starch content (70–80%) in unfermented cassava peels, which form a gel when their suspension is heated together with water, as reported by Rashwan et al. (2024). Like the amylose, on fermentation also there is a reduction in the entropy as enzymatic activity decreases, which leads to a lower content of amylopectin. As a result, amylopectin, being highly branched, is degraded at different rates compared to linear chains of the glucose that makes up amylose.

For the cyanide content of cassava peels, it was found to be in the range of 0.80 to 14.90 ppm. The cyanide content of unfermented cassava peels is greater than that of their fermented form. According to Oloya et al. (2024), cyanide levels in unfermented cassava peels can be relatively high, and the amount varies with a factor such as cultivar; bitter varieties contain higher cyanogenic content than sweet ones. Generally, fermentation reduces the cyanide content of cassava peels. This process results in the release of hydrogen cyanide, which either volatilises and is released into the air or further decomposed by microorganisms into forms that are less toxic. The decrease in cyanogenic glycosides during fermentation is mainly dependent on the length of time for which fermentation occurs, temperature and pH, as well as the microorganisms involved. Hence, the necessity for effective fermentation of cassava peels in order to pool down cyanide levels within acceptable limits. Moreover, focusing on these variables will further aid in fine tuning the fermentation process to achieve maximum reduction of cyanide.

## Conclusion

The results indicated that cassava peels were rich in dry matter and had low protein content, whereas the effluents contained a high level of biochemical oxygen demand (BOD) and chemical oxygen demand (COD). The results indicated that the majority of the physicochemical components of both cassava peel and effluents released into the environment surpass the permissible thresholds established by FEPA/WHO for all classifications of industrial effluent discharge. Consequently, the findings of this study indicated the necessity for regulatory agencies to ensure adherence to established criteria to safeguard both human health and the environment. The results also showed that fermentation increases the bioavailability of nutrients in cassava peels and enhances their nutritional value. Although its effects on some of the parameters evaluated were not significant, implementing proper waste disposal methods and treatment technologies is thus recommended to help mitigate the negative impact of cassava processing on local ecosystems, prevent environmental pollution, and maximise resource utilisation. Sustainable waste management strategies, including composting, biogas production, and wastewater treatment, should be adopted to mitigate negative ecological effects. Recommendations for policymakers and governing authorities for the establishment of guidelines and limitations for the appropriate discharge of industrial effluents should be developed.

## Data Availability

All data associated with this study are reported in the manuscript and no other data are deposited in any public or closed database.
